# The Water to Land Transition Submerged: Multifunctional Design of Pectoral Fins for Use in Swimming and in Association with Underwater Substrate

**DOI:** 10.1093/icb/icac061

**Published:** 2022-06-02

**Authors:** Melina E Hale, Seth Galdston, Benjamin W Arnold, Chris Song

**Affiliations:** Organismal Biology and Anatomy, University of Chicago, 1027 E. 57th St., Chicago, IL 60637, USA; Organismal Biology and Anatomy, University of Chicago, 1027 E. 57th St., Chicago, IL 60637, USA; Organismal Biology and Anatomy, University of Chicago, 1027 E. 57th St., Chicago, IL 60637, USA; Organismal Biology and Anatomy, University of Chicago, 1027 E. 57th St., Chicago, IL 60637, USA

## Abstract

Fins of fishes provide many examples of structures that are beautifully designed to power and control movement in water; however, some species also use their fins for substrate-associated behaviors where interactions with solid surfaces are key. Here, we examine how the pectoral fins of ray-finned fish with these multifunctional behavioral demands, in water and on solid surfaces, are structured and function. We subdivide fins used in swimming and substrate contact into two general morphological categories, regionalized vs. generalized fins. Regionalized fins have ventral rays that are free from connecting membrane or in which that membrane is reduced. Dorsally they maintain a more typical membranous fin. While all pectoral fins vary somewhat in their morphology from leading to trailing edge, generalized fins do not have the substantial membrane loss between rays that is seen in regionalized fins and the distal edge anatomy changes gradually along its margin. We add a new case study in regionalized fins with the dwarf hawkfish (*Cirrhitichthys falco*). Hawkfishes are most often found perching and moving on structures in their environments. During perching, the free ventral rays are in contact with the substrate and splayed. We found that unlike other fish with regionalized pectoral fins, hawkfish maintain use of the dorsal membranous region of its pectoral fin for rhythmic swimming. We found that typically hawkfish bend their ventral free rays under, toward the medial hemitrichs or hold them straight during substrate-associated postures. This appears also to be the case for the ventral free rays of other species with regionalized fins. Generalized fin use for substrate contact was reviewed in round gobies (*Neogobius melanostomus*). In addition, although their lobe fins are not representative of ray-finned fish anatomy, we explored fin contact on submerged substrates in the Senegal bichir (*Polypterus senegalus*), which has a generalized distal fin (no free fin rays or distinct membrane regions). Both groups use their pectoral fins for swimming. During substrate-based postures, unlike hawkfish, their distal rays generally bend outward toward the lateral hemitrichs and a large swath of the fin membrane can contact the surface. The alternative demands on multifunctional fins suggest specialization of the mechanosensory system. We review mechanosensation related to fin movement and surface contact. These alternative regionalized and generalized strategies for serving aquatic and substrate-based functions underwater provide opportunities to further investigate specializations, including sensory structures and systems, that accompany the evolution of substrate-based behaviors in vertebrates.

## Introduction

For many species, movement and posture in day-to-day lives require interaction with different physical environments: air and land (e.g., bats that fly and perch), air and water (e.g., birds that fly and paddle), and water and land (e.g., frogs that swim and hop). Locomotion across the last of these environment pairs, water and land, has been a focus of research for insights into the evolution of terrestrial vertebrates and walking from aquatic forebearers and their fin-based swimming.

While movement related to the "water to land transition" generally implies movement on land that is above the water surface, the sea-, river-, or lakebed also provides solid substrate, underwater land, with which animals interact. Among fishes, there is a wide range of species that routinely associate with underwater substrate and also swim as part of their normal behavioral repertoire. Many more shelter on or in underwater structures to sleep, indicating regular substrate contact, even when their awake behaviors are generally water-based.

Strategies that fish use to interact with the fluid environment and submerged substrates range from distributing functions among body elements to multifunctionality of a single structure. In the former category, examples include skates that use pelvic fins to punt and pectoral fins to swim (e.g., Koester and Spirito 2003; Di Santo et al. 2017), and African lungfish that use their pelvic fins to walk at slow speeds and axial bending for swimming (e.g., King et al. 2011). The division of labor between body elements may facilitate specialization of anatomy due to reduced competing functional pressures on one element.

Here, we examine multifunctionality of a given body element focusing on ray-finned fishes. We investigate how the paired pectoral fins are able to be multifunctional for swimming and for substrate associated postures. The pectoral fins are commonly used in swimming of many fish groups. Many species use labriform locomotion, swimming driven exclusively or highly dominated by pectoral fin propulsion (e.g., Walker and Westneat 1997; Drucker and Lauder 2000). Others coordinate pectoral fin movement with movement of median fins and/or the body axis (e.g., Arreola and Westneat 1996; Hove et al. 2001). Still others do not beat the pectoral fins for propulsive force generation but use them as planes to generate lift during swimming powered by the body axis (e.g., sculpins; Taft et al. 2008) or to help angle the body in the water during swimming (e.g., sturgeons (Wilga and Lauder 1999) and sharks (Wilga and Lauder 2000)).

Many fishes accomplish substrate-based behavior with pectoral fins that are also used in swimming. These approaches can be binned into two categories. Some species have fins that are strikingly regionalized with dorsal and ventral sections appearing to serve either primarily swimming or substrate-based function, whereas others have fins that appear relatively undifferentiated from leading to trailing edge.

We examine these two approaches to multifunctionality of the pectoral fins in water and on underwater substrate. Fluids and solid surfaces are very different sensory environments; we begin with an overview of what is known about fin sensation with substrate contact and for movement in water. We then turn to the general categories of multifunctional regionalized and generalized fins. For regionalized fins, we delve into their evolution, finding that extreme pectoral fin regionalization arises independently in the sister orders Perciformes and Centrarchiformes, with different associated changes in swimming function. We review literature on swimming and multifunctionality of pectoral fins and provide a new example with hawkfishes, which have strikingly regionalized pectoral fins. We add data and observations to add to the research on bichir pectoral fin function that has been studied in swimming and terrestrial movement.

We aim for this review and these new data to highlight the importance of multifunctionality of the pectoral fins and the need to consider even more broadly the behavioral use of a body element when assessing the relationship between structure and function. In addition, the body of work explored suggests alternative design solutions for multifunctional mechanical structures.

### Mechanosensation for fin use on solid substrate and in water

Mechanosensitivity of the pectoral fins has been reported in a range of species (e.g., Morrill 1895; Lowenstein 1956; Bardach and Case 1965; Ono 1979; Finger 1982, 2000; Silver and Finger 1984; Williams et al. 2013; Hardy et al. 2016; Aiello et al. 2017), suggesting that these capabilities are found across the phylogeny of fishes. Here, we review work that bears on understanding of fins that are contacting substrate.

Sea robins, the best-known example of regionalized pectoral fins with ventral free rays, are known to have mechanosensitive rays. In their seminar study, Silver and Finger (1984) found that sea robin fin ray nerves responded to touch and were proprioceptive, with different units generally responding to movement or position of the rays. Further detailing of mechanosensation, and association of physiology with peripheral neuroanatomy in free rays, would be an exciting addition to understanding of sea robins. Especially with their use of free rays to actively probe the bottom substrate, sea robins are a fascinating model for ray specialization and regionalization.

**Fig. 1:**
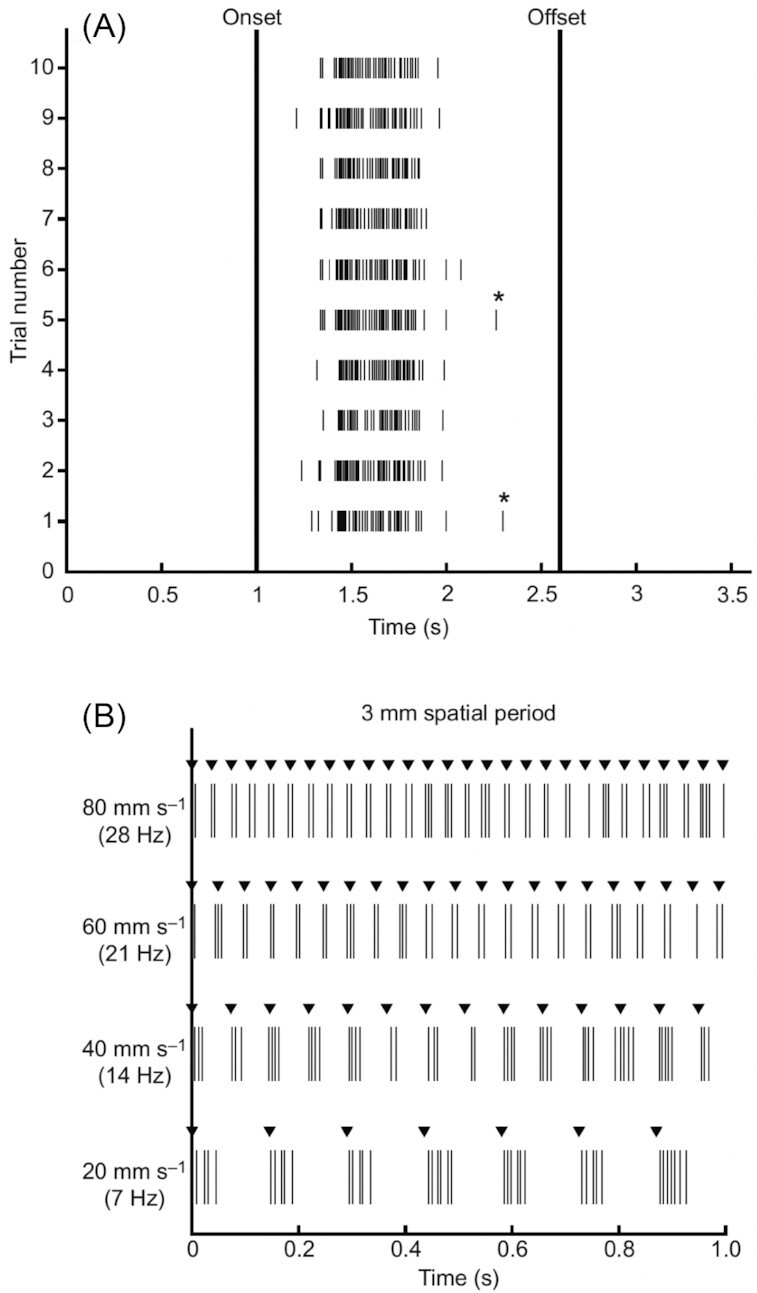
Touch sensation in the round goby, *Neogobius melanostomus*. Raster plots show spiking of afferent nerve fibers in response to fin ray stimulation. **(A)** A total of 10 trials of proximodistal brushing along an 8-mm length of a ray at 5 mms^−1^, passing over an afferent ending showing consistency of the response. **(B)** A drum with a 3-mm spatial grating rotating so that the gratings make light, brief contact with the fin, brushing proximodistally. Recordings are shown for at four speeds. The ability of the sensory afferent to sense the structure of the stimulus through this range of speeds (drum rotation rates) indicates that these fibers can faithfully report on fine substrate features. Asterisks indicate spikes excluded from analysis of spike bursts as they did not fall within the threshold for firing rate. These data show the fine spatial and temporal sensitivity and reliability of touch sensors in the pectoral fin. Reprinted from Hardy and Hale (2020)

The pectoral fins of the round goby, non-regionalized, substrate-contacting fins, demonstrate touch capability for sensing fine features of contacted substrates. By recording activity of fin ray afferent nerve fibers as a probe brushed along the fin membrane, Hardy and Hale (2020) showed fine spatial and temporal resolution of mechanosensors in the fin membrane (Fig. 1). The receptive fields recorded were several millimeters in diameter and their response properties were consistent with those previously described in mammals; increasing scan speeds increased the rate of firing and decreased the duration of the activity and overall number of spikes recorded (Hardy and Hale 2020). Pressure on the skin in a single location (not brushed along it) showed responses characteristic of slow adapting afferents in other species: high spiking at the onset of the stimulus followed by prolonged activity during continued application of force. Repeated stimulation of the skin through presentation of a rotating drum textured with gratings found that afferent activity phase locked to the periodic stimulation across a range of speeds, indicating the ability of afferents to sense fine features of contacted surfaces (Fig. 1). These data suggest that during substrate contact, detailed mechanical characteristics of the touched surface can provide input that may be used to adjust fin or fin ray posture and position.

Proprioceptive sense is also well-developed in membranous pectoral fins and provides feedback during fin bending. While not specifically studied in round gobies, proprioception is well-known from a range of other teleost species (e.g., Williams et al. 2013; Aiello et al. 2017) and thought to be common to all. As detailed in the bluegill sunfish (Williams et al. 2013), fin ray afferents sense movement of fin rays, with afferent activity reflecting the speed, amplitude, and duration of movement. In prolonged bends of the fin rays at higher amplitudes, continuous spiking has been observed providing input through the period that the bend in the fin is held. These data indicate that there is mechanosensory feedback throughout the swimming fin stroke and likely when fins are bent for extended periods during substrate associated postures. Unlike in sea robins (Silver and Finger 1984), there was no indication that different units responded to movement and to position in bluegills or other species (Aiello et al. 2017), suggesting potential specialization in free fin rays or fins that commonly interact with the bottom substrate or in sea robins.

Testing the role of mechanosensory feedback in fin movement, transection of fin ray nerves of the bluegill sunfish resulted in changes to fin movement, particularly increased frequency and decreased amplitude of the fin strokes in hover (Williams and Hale 2015). Comparable transections and tests of steady swimming of parrotfish found similarly increased fin beat frequency and changes to the fin stroke, notably stroke plane angle (2020). In addition, fish transitioned from fin-driven swimming to *axial* swimming at lower speeds, possibly due to decreased efficiency of the fin strokes without mechanosensory modulation. To our knowledge, no comparable experimental manipulation has been performed for free rays or for more typical fins in functions related to surface contact.

Experiments manipulating other sensory modalities also add to our understanding of the mechanosensory function of the fins. Flammang and Lauder (2013) swam bluegill sunfish through a peg board obstacle course before and after visual and lateral line inputs were impaired. Bluegills more frequently tapped their fins on pegs under the experimental conditions, though some touching occurred in all conditions. Flammang and Lauder suggest that mechanosensory feedback can help compensate for loss of other senses as fish navigate this complex environment. Another study, Windsor et al. (2011), examining cavefish that do not have visual ability but do have lateral line, found that pectoral fin touch seems not to play a significant role compared to lateral line feedback for swimming. Across the fishes, many species live in environments with limited visibility. In addition to caves, turbid water and deep oceans restrict visibility and many species are nocturnal. Fin ray proprioception may play a larger role in providing feedback about surrounding structure and posture in those groups but its relationship to lateral line function, particularly for object navigation, would be critical to assess.

The repertoire of pectoral fin movement and touch and proprioception are diverse and have only begun to be explored (but see: Bardach and Case 1965; Moreno 1980; Gladstone 1994; Pace and Gibb 2009; Ferrando et al. 2014; Lönnstedt et al. 2014; Hardy and Hale 2020; Hale 2021). Many questions on the specialization of mechanosensation for behavior of fish have yet to be addressed. It is known, however, that there is variation in fin mechanosensation among species and that variation reflects fin mechanics and function. Aiello et al. (2017) demonstrated neuromechanical tuning of mechanosensory responses in species of wrasse and parrotfish (family Labridae). Examining multiple closely related species pairs that included a stiff finned and flexibly finned species, it was found that the threshold for physiological response to fin bending occurred at lower bending amplitudes in the stiff finned compared to flexible finned species. Since stiff fins operate at lower bend amplitudes than flexible fins, it was postulated that mechanosensitivity is tuned to the relevant operating range of fin ray bending during behavior. It is highly likely that there are sensory specializations for substrate associated fins that have particular roles for touch and proprioception in their routine activity.

In the following sections we focus on fin gross morphology and movement. Diversity among fish species that use their fins both in water and on underwater surfaces raises questions about related adaptations in the fin's mechanosensory system and provides a testbed for studying them.

## Regionalized multifunctional fins

Regionalized pectoral fins offer some of the most striking and beautiful examples of regionalized limb specialization in vertebrates; as mentioned above, sea robins (genus *Prionotus*) provide the quintessential example. The ventral region of the sea robin's pectoral fin includes three free fin rays, rays that are not connected by fin membrane, while the dorsal region is a broad, often colorful, fin membrane invested with soft rays. The dorsal region is thought to be involved in social signaling. It is not known to play a locomotor role during swimming and sea robins do not appear to actuate the membranous region of the pectoral fins rhythmically during movement. The ventral rays are known to be chemo- and mechano-sensory (e.g., Bardach and Case 1965; Silver and Finger 1984). They are used in foraging (e.g., Bardach and Case, 1965; Davenport and Wirtz 2019) and are thought to be locomotor (e.g., Renous et al. 2000). The ventral free rays are actuated by pectoral fin muscles that show a range of specializations (Petersen and Ramsay 2020), including hypertrophied abductor and adductor superficialis muscles. For comparison to other taxa, Petersen and Ramsay (2020) termed these muscles, respectively, the walking ray levators and superficial walking ray retractors.

There are multiple evolutions of ventral ray specialization in fishes. Focusing on the Perciformes (e.g., Betancur et al. 2017; Smith and Busby 2014; Smith et al. 2018), sea robins belong to the family Triglidae in that order, which also includes other families with fin regionalization and multifunctionality. In addition to the Triglidae, the Scorpaenidae, scorpionfishes and their relatives, and Cottoidei, the suborder that includes sculpin families (Smith and Busby 2014), are other groups with regionalized fins and substrate-associated pectoral fin behavior that are in the order perciforms. Not all groups in the Perciformes have such clearly regionalized fins. For example, the family Serranidae that includes the sea basses and groupers have more typical fins with membrane connected rays along the full span of the pectoral fins. We note that recent phylogenies on some of these key groups (Smith and Busby 2014; Smith et al. 2018) provide additional detail on classification and relationships that could not be covered here.

Ventral ray specializations are not unique to the Perciformes. In fact, the Centrarchiformes, sister order to the perciforms also includes striking examples of multifunctional, regionalized fins. The hawkfishes (family Cirrhitidae) and their relatives have specialized ventral fin rays that are at least as extreme, if not more extreme, than those of sea robins. Like the Perciformes, regionalization is not ubiquitous within the order. Chubs (*Kyphosus*), sunfishes (*Lepomis*), and other species in the centrarchiforms have more typical fins. The bluegill sunfish, *Lepomis gibbous*, discussed previously, is a well-known model of pectoral fin use in swimming and hovering with a membranous, unregionalized fin.

**Fig. 2:**
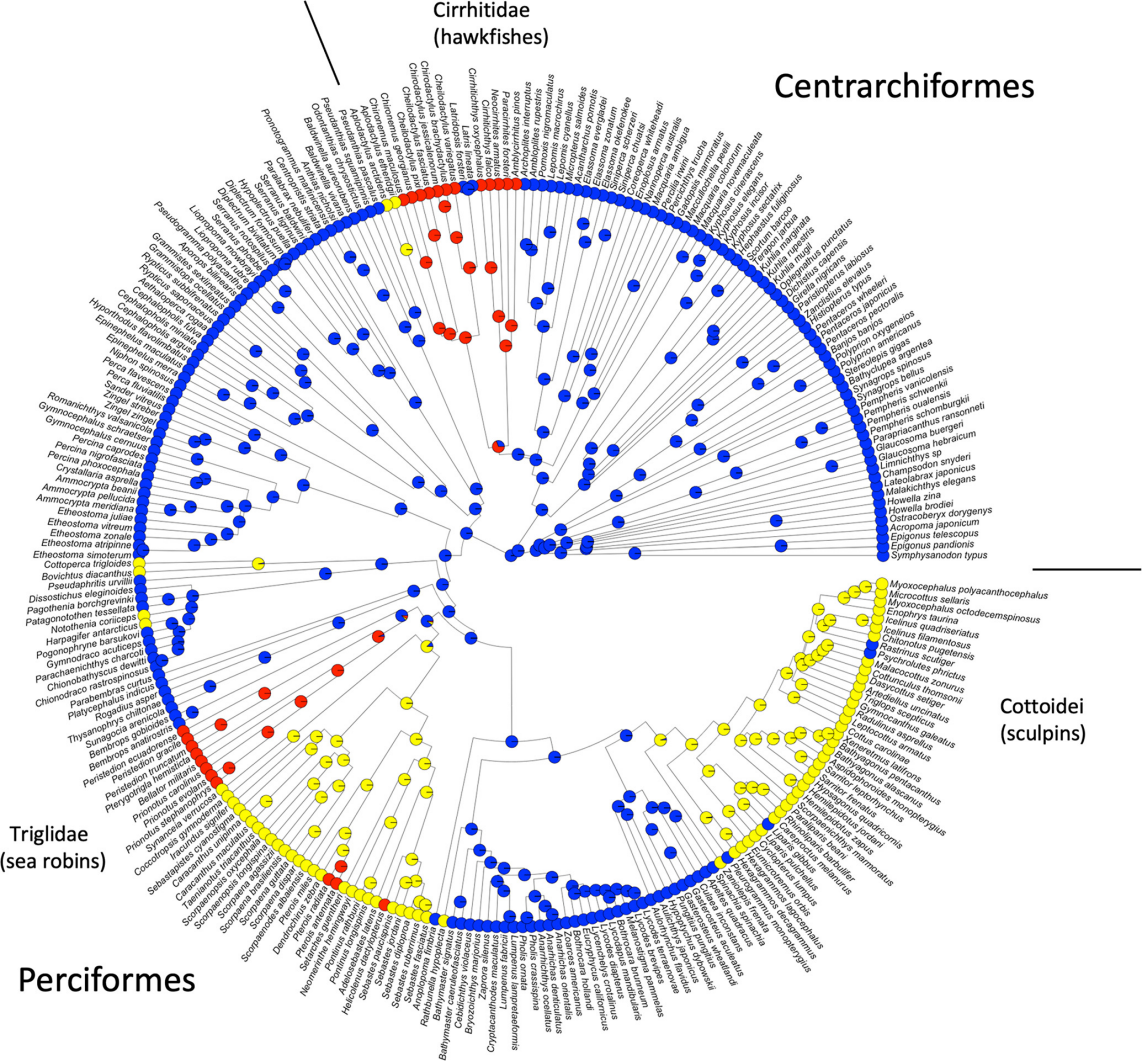
Mapping pectoral fin regionalization on a phylogeny of the group that includes orders Centrarchiformes and Perciformes, highlighting the families discussed, the Cirrhitidae (hawkfishes), Triglidae (sea robins), and Cottoidei (sculpins). Extreme ventral regionalization (near complete loss of membrane between rays) is shown in red. Moderate ventral regionalization (partial loss of membrane connecting ventral rays) is shown in yellow and minimal or no loss of membrane between ventral rays is shown in blue. Note that the hawkfishes are in the centrarchiforms and independently evolved ventral fin regionalization from the other families shown, members of the Perciformes. Those two groups also appear to have evolved ventral regionalization independently. Phylogeny from Betancur et al. (2017), but also see Smith and Busby (2014) and Smith et al. (2018)

We mapped the ventral pectoral fin regionalization on a recent phylogeny of fishes (Fig. 2) to understand whether distinctive, highly specialized pectoral fin regionalization was independently evolved in each order or the basal condition of their common taxonomic grouping, inclusive of Perciformes and Centrarchiformes and no other orders. Methods are included at the end of the paper and Appendix 1 lists species and sources. Hawkfishes evolved extreme ventral regionalization with free fin rays independently of sea robins. Within the perciform it also appears that sea robins and sculpins independently evolved ventral regionalization. There are numerous other species with ventral pectoral fin specialization shown in Fig. 2 that are outside of the scope of this paper but that point to the rich diversity of pectoral fin regionalization and specialization within this two-order group and, additionally, taxa where specialization appears to have evolved and then been lost. Other substrate associated species, like the perciform group of darters (Etheostomatinae), are known to station hold well in flow using the pectoral fins (Carlson and Lauder 2010). While their fins do not have the striking anatomy of those coded as specialized and regionalized here, there may be more subtle and/or graded difference in pectoral fin morphology from dorsal to ventral that is associated with this use of the fins. More broadly, closer examination of specimens than was possible here will surely add detail and richness to this broad-strokes survey.

Below we explore multifunctionality and regionalization in independent evolutions within the perciform and centrarchiform fishes. In the perciforms, studies on the sculpins have directly addresses pectoral fin function and regionalization. We also add new data and observations on a centrarchiform species, the dwarf hawkfish (*Cirrhitichthys falco*).

### Ventral pectoral fin regionalization in Perciformes: sculpins

Sculpins (Cottoidei) are a fascinating benthic fish species that live in a range of environments, often in flow, and have long been known to use their pectoral fins to hold station (Webb 1989). As described in the father lasher (*Myoxocehpalus scorpius*), during station holding against substrate, the ventral fin rays extend and contact the substrate, often forward of the fin base. The more dorsal fin membrane extends back to lay against the side of the body and is thought to provide streamlining (Webb 1989). A total of two studies (Taft et al. 2008; Kane and Higham 2012) have further investigated pectoral fin regionalization and function. The first (Taft et al. 2008) examined function of dorsal and ventral pectoral fin regions in a low flow environment with behavioral experiments, focusing on a congeneric, the benthic longhorn sculpin, *Myoxocephalus octodecimspinosus*. The second (Kane and Higham 2012) examined fin morphometrics from a range of cottids and explored the relationship between pectoral fin anatomy and the flow environment naturally experienced by each species.

The benthic longhorn sculpin demonstrates different use of dorsal and ventral fin regions during both station holding and swimming behaviors (Taft et al. 2008). During station holding, the ventral pectoral fin rays are splayed and contact the bottom surface of the tank, anterior to the dorsal region of the fin, and are believed to be to be gripping the bottom. The dorsal membranous region of the fin is positioned above the bottom surface and angled toward the body, perhaps playing a role in streamlining as considered for the father lasher. At higher speeds, rhythmic swimming was driven by the median fins, caudal fin and axial bending (the father lasher was also noted to use the body axis and caudal fin in swimming [Webb 1989]). Rather than tucking the full pectoral fin against the body wall, as is common with axial-based swimming, the ventral rays of the benthic longhorn sculpin were tucked but the dorsal rays and membrane splayed laterally. Taft et al. (2008) suggest that this surface may provide lift to offset the negative buoyancy of sculpins.

Among sculpins, species in higher flow environments where higher gripping performance for station holding would be expected to be more important, have more pronounced regionalization (Kane and Higham 2012). In sculpins, the extent of the membrane between the ventral rays varies among species. While their rays are not fully "free" as seen in the sea robins and hawkfishes, the distal free tips of the rays extend beyond the membrane connected region. Using multivariate analysis approaches to examine association between flow environment and fin morphology across nine species, Kane and Higham (2012) found that a group of species thought to have greater need for gripping in flow have relatively less membrane connecting the ventral rays.

In summary, the full pectoral fin of the sculpin appears to function in swimming and station holding, with subdivision of function between the dorsal and ventral regions. Specialization of the ventral rays for gripping, particularly pronounced in higher flow environments, is associated with ventral fin ray morphology that shares features with other benthic species that have regionalized fins for substrate-based posture: notably less membrane connection between the rays (Kane and Higham 2012). The dorsal region of the fin, while functioning in swimming, does not beat rhythmically to generate propulsive force but, at least in some species, does engage during swimming, extending laterally and likely serving as a lift generating surface (Taft et al. 2008).

### Ventral pectoral fin regionalization and function in the hawkfishes

The hawkfishes (Cirrhitidae) are benthic reef fishes. They lack a swim bladder, as is common in benthic fish species (McCune and Carlson 2004). Hawkfish may experience waves and currents in their reef habitats and use structure in the environment to shelter from predators and from which to attack prey (DeMartini 1996). The name hawkfish comes from the hawk-like perching of these species. Here, we examined the dwarf hawkfish (*C. falco*). The pectoral fins of *C. falco* were described by Randall (1963). They have 14 rays in each pectoral fin, as do confamilials. The dorsal eight rays are connected by membrane. Of those eight all but the leading edge ray are branched. The ventral six rays are unbranched free rays. The second of these is the longest, measured by Randall to be 1.6 times the length of the dorsal, branched rays. Free ventral rays are likely important for maintaining a fixed posture in prey capture behavior and to help avoid visual detection by predators.

Here, we briefly describe the movement of the pectoral fins in swimming and perching of *C. falco* to explore the use of the dorsal membranous region of the fin and to begin to quantify the use of the ventral rays in perching and assess their involvement in swimming. Our primary question regarding hawkfish swimming was a simple one: Do the pectoral fins beat during slow swimming? We also ask the question: Do movements of the dorsal and ventral regions of the pectoral fin differ during swimming? Another fundamental question: Are ventral rays used in perching? Has been addressed previously with a clear 'yes' in natural history and taxonomic work (e.g., Randall 1963; Randall et al. 1990). Here, we provide additional quantification of perching in a simple tank environment. Specific questions of interest include: Overall, how are the rays contacting the surface—bending outward or under? Do hawkfish vary ventral ray posture in our simple experimental conditions? If so, how is that related to body position? We also provide preliminary observations on the morphology of ventral fin ray musculature.

**Fig. 3:**
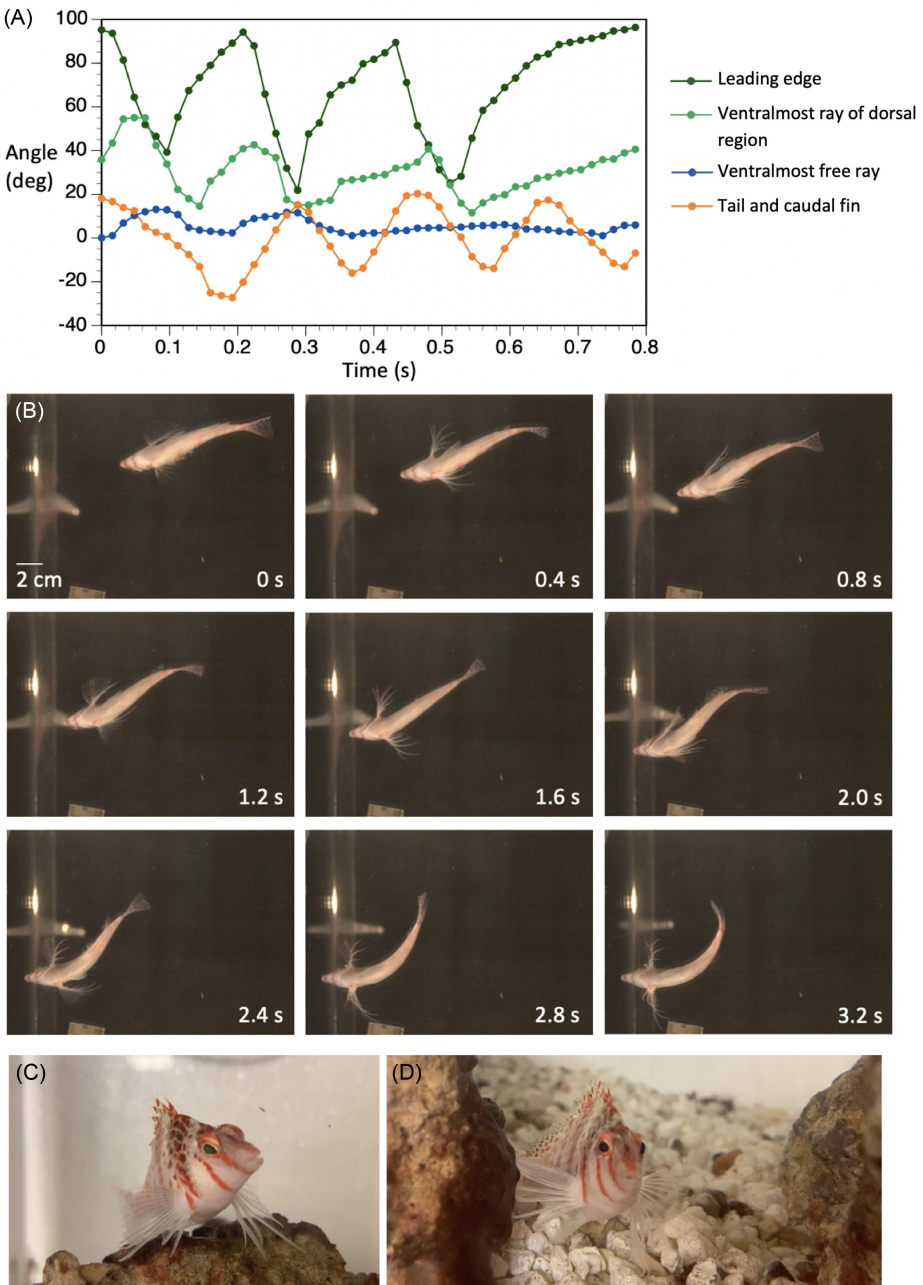
Pectoral fin swimming and perching in the dwarf hawkfish, *C. falco*. **(A)** Three cycles of swimming showing angular movement of landmarks on a pectoral fin (left) and the body axis. The leading edge and membranous region of the fin are actuated rhythmically. There is some coincident movement of the ventral free rays but that movement is irregular. **(B)** A trial of hawkfish swimming and perching (not the same trial as in **A**) illustrating movement of the membranous dorsal region of the pectoral fin and ventral free rays. Successive instances of peak abduction of the dorsal region of the pectoral fin can be seen during swimming at time 0 and 1.2 s, illustrating that the fins beat synchronously (in phase). At times 2.8 and 3.2 s, the free rays are splayed during perching. **(C)** and **(D)** Images of a hawkfish in its home aquarium showing perching on irregular substrate. Fin rays bend back toward the medial aspect of the fin and are curved more or less distally, accommodating varied distances to the surfaces beneath them

Hawkfish do beat their pectoral fins during swimming. In our recordings pectoral fin movement was always performed in concert with the caudal fin beats and always with symmetrical abduction and adduction between the left and right pectoral fins. This pattern can be seen in Fig. 3(A) where the leading edges of both fins reach peak abducted at 0 and 1.2 s frames. Fin beat cycles were measured from peak abduction to peak abduction of the leading edge. Figure 3(B) shows angular movement, measured from ventral view, of a pectoral fin's leading edge, the ventralmost fin of the membrane bound dorsal region, the most ventral free ray and the caudal fin to illustrate differences in amplitude of the fin's regional abduction and coordination. From analysis of leading-edge movement, the frequency of fin beats was 3.66 ± 0.95 beats per second, peak abduction was 94° ± 8°, and overall angle of the fin stroke was 75° ± 14°. The ventralmost ray of the dorsal region also beat but trailed the leading edge temporally and had a lower stroke angle (Fig. 3); peak abduction was 46° ± 13°, overall angle of the fin stroke was 38° ± 16°. The average angle of the most ventral free fin ray was 29° ± 13°. There was little movement of the ventral free rays during swimming and no clear cycles of abduction and adduction of the free ray digitized.

**Fig. 4:**
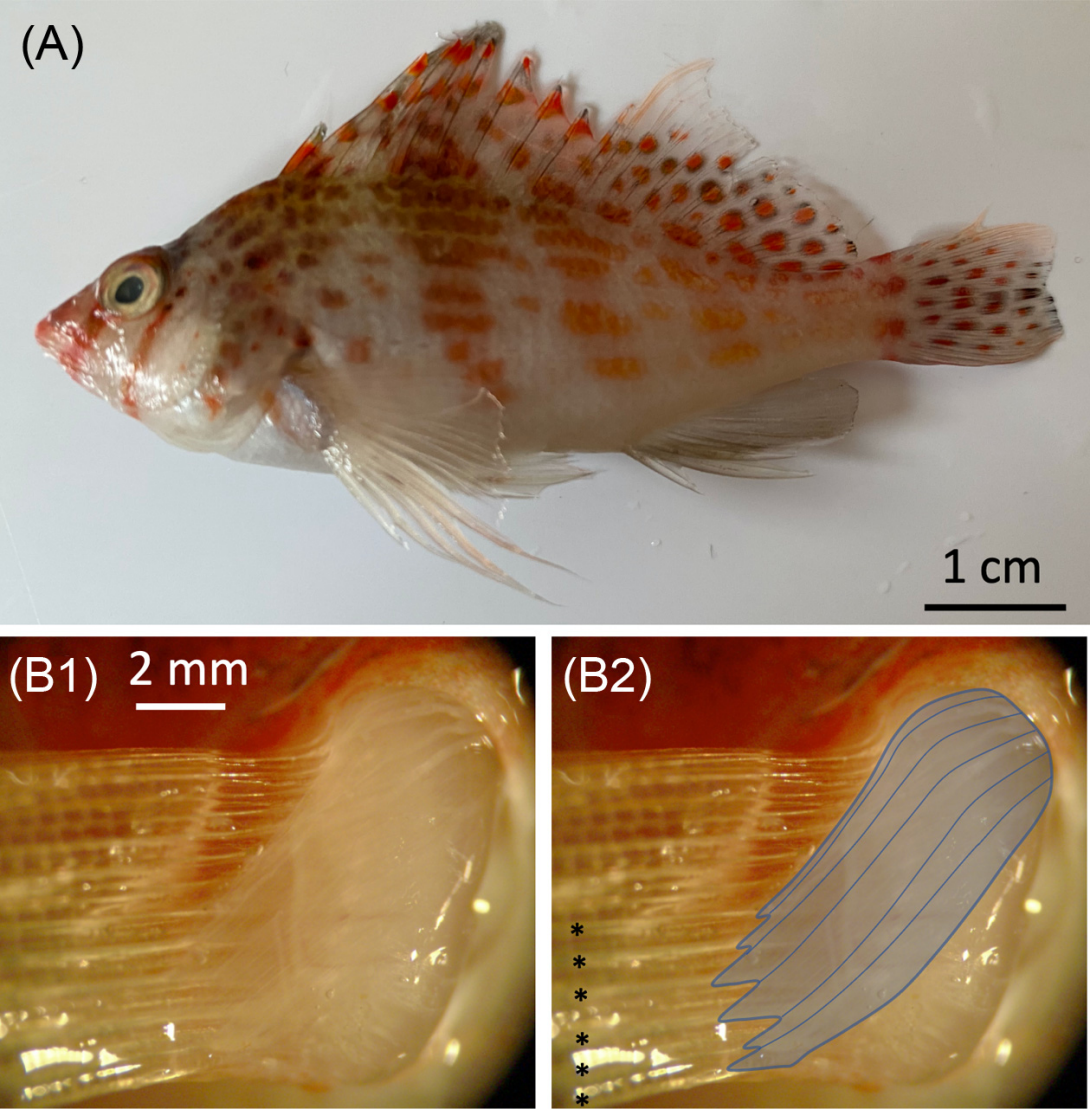
Pectoral fin musculature of the dwarf hawkfish, *C. falco*. **(A)** The two regions of the pectoral fin: dorsal region with membrane-connected rays and ventral region that consists of free rays. (**B1**and **B2**) Adductor superficialis muscles of the ventral free rays are large and insert distal to the base of the rays (muscles outlined in **B2**). Ventral free rays are indicated with asterisks

During perching on the horizontal tank floor in our experimental conditions, hawkfish used ventral fin rays to lift their head and anterior body off the tank floor (Fig. 3), with the snout at 2.17 ± 0.27 cm above the bottom. Rays were curved back toward the medial side of the fin at contact such that the distal ends of the rays lay on the bottom surface with the lateral aspect of the rays down. This curving back is consistent with fin ray bending of sea robins and sculpins. Hawkfish splayed their ventral fin rays with the most ventral ray being closest to the body and the others spreading in sequence from there. (Ventralmost [Free Ray 6 (FR6)]: 1.56 ± 0.27 cm; FR5: 2.04 ± 0.27 cm; FR4: 2.69 ± 0.20 cm; FR3: 3.53± 0.35 cm; and FR2: 4.22 ± 0.19 cm). The first free ray commonly overlapped the others and could not be measured reliably. Curvature of the rays toward the medial aspect of the fin and splaying of the fin rays were also seen in hawkfish in their home tanks when perching on the more irregular substrates (Fig. 3), as shown in these examples, posture and fin splay vary markedly. The behavioral use of the ventral rays raised question of their control and whether muscle enlargement and specialization, as occurs with free fin rays in *Prionotus*, is also present in hawkfish. While not fully described here, we share the observation that abductor and adductor superficialis pectoral fin muscles associated with the ventral free rays are hypertrophied and angled sharply toward the rays. This is shown for the adductor superficialis in Fig. 4. Insertion points on the rays are distal to the base of the ray (Fig. 4), perhaps increasing leverage and control. These observations that the muscles of the ventral rays are relatively large aligns with our assessment that these rays are actively used in substrate-related behaviors and important to control of substrate-based posture.

The independently evolved regionalized fin of the hawkfishes shares features of substrate-based posture and associated anatomy with other taxa described. Ventral free rays splay across the contacted surface, extending forward of the fin base generally bending inward, toward the medial aspect of the fin. Contrasting between the groups is the use of the dorsal region in swimming, with hawkfish retaining typical pectoral fin beating in locomotion while sculpins and sea robins appear to have lost rhythmic fin beating but may use the pectoral fins in other roles during swimming, such as lift generation (Taft et al., 2008).

## Generalized multifunctional fins

While all pectoral fins have some asymmetry from leading to trailing edge, here we categorize as having "generalized" fin morphology, those pectoral fins that lack notable features of the regionalized fins described above, specifically, marked lengthening of ventral rays and loss of substantial membrane between the ventral rays.

A number of fish groups known for using fins on substrates have pectoral fins with a generalized distal morphology; two of those groups are the gobies and bichirs. Despite this behavioral similarity, these groups are very different. Phylogenetically they are distantly related, with gobies found well within the teleosts in the order Gobiiformes while bichirs are in the order Polypteriformes, the most basally branching lineage of ray-finned fishes. With some exceptions (e.g., Winterbottom, 1976), both goby and bichir pectoral fins have a continuous distal membrane, but their fin bases differ. Goby fin rays extend from a base at the body wall, as is typical of teleosts, including those with a regionalized fin morphology described above. Bichir pectoral fins have a fin base that extends farther from the body wall and moves with the fin membrane and rays, which flare around the distal margins (e.g., Dean 1910; Wilhelm et al. 2015). By focusing on these two examples, we review and further explore how very different generalized fins are used in underwater substrate association.

### Fin swimming and underwater postures in gobies

Gobies are generally found underwater perched on sand, mud, rocks, or peeking from shelter, such as crevices in rocks or holes in sand or mud. In some cases, their association with substrate is extreme, such as that seen in the waterfall climbing gobies (e.g., Schoenfuss and Blob 2003) and mudskippers traversing air-exposed mud flats (e.g., Pace and Gibb 2009; Kawano and Blob 2013). While we are focused on the pectoral fins here, we note that other body elements are important in these behaviors, for example the oral sucker and pelvic fins drive waterfall climbing (Schoenfuss and Blob 2003).

Locomotor behavior in the round goby has received outsized attention as part of efforts to control this invasive species. In particular, swimming performance has been a focus of considerable work (e.g., Hoover et al. 2003; Tierney et al. 2011; Pennuto and Rupprecht 2016). Round gobies have large, rounded, and flexible fins. Pennuto and Rupprecht (2016), in a flume study examining round goby swimming at a range of speeds, found pectoral fin-based swimming to be common at slow swimming speeds (10 cm s^−1^ for fish 40–130 mm in length). The use of pectoral fins in substrate associated station holding in flow was noted by Tierney et al. (2011), suggesting that in flow environments the pectoral fins create force that helps the fish remain against the bottom, augmenting gripping suction generated by the pelvic fins. In an examination of pectoral fin contact with substrate (but not in flow) underwater in the round goby, pectoral fins were found to use the distal half of the fin along its full leading to trailing edge margin against surfaces (Hardy and Hale 2020). The fin was also shown to conform to the shape of the structure that it contacts, whether that surface is a flat surface below the fish, or a vertical wall or curved horizontal surface adjacent to it. These studies illustrate multifunctionality of the round goby's generalized pectoral fins in swimming and underwater posture. It is likely that this multifunctionality is common in the order Gobiiformes; the barred mudskipper (*Periopthalmus argentilineatus*), in a different family of gobies from the round goby, has also been shown to swim as well as move on mudflats with its pectoral fins (Pace and Gibb 2009).

### Fin swimming and underwater postures in bichirs

The Senegal bichir (*Polypterus senegalus*) has been the focus of a set of terrific studies on locomotor behavior in aquatic and terrestrial environments (e.g., Standen et al. 2014, 2016; Du and Standen 2017, 2020). We review just a small portion of that work, results that focus on aquatic behavior. We add observations on substrate-associated posture underwater.

Senegal bichirs use their pectoral fins for slow swimming underwater. Standen et al. (2016), found that during slow swimming (under 0.5 BL s^−1^), the pectoral fins were the dominant source of propulsive force, with minimal concurrent movement of the body axis or caudal fin. The pectoral fins beat rhythmically in slow swimming and the left and right fins were abducted and adducted in phase (Standen et al. 2016). Pectoral fin swimming kinematics of animals examined here were consistent with results of Standen et al. (2016). Standen et al. (2016) reported very short stroke durations, in the range of 40–60 ms and ours were a little slower (71.8 ms ± 11.5 ms). This duration, indicating a fin beat frequency of around 14 Hz, seems very high. However, we also note that the angle of fin movement is quite low (24.3° ± 11.3°). Unlike their fin-engaged walking behavior above water, we did not observe bichirs in water moving along the floor of the filming tank using their fins. This may be due to the surface properties of the glass, but we also note that we have not seen substrate-based movement home aquaria that contain both sandy and rocky submerged substrates.

**Fig. 5:**
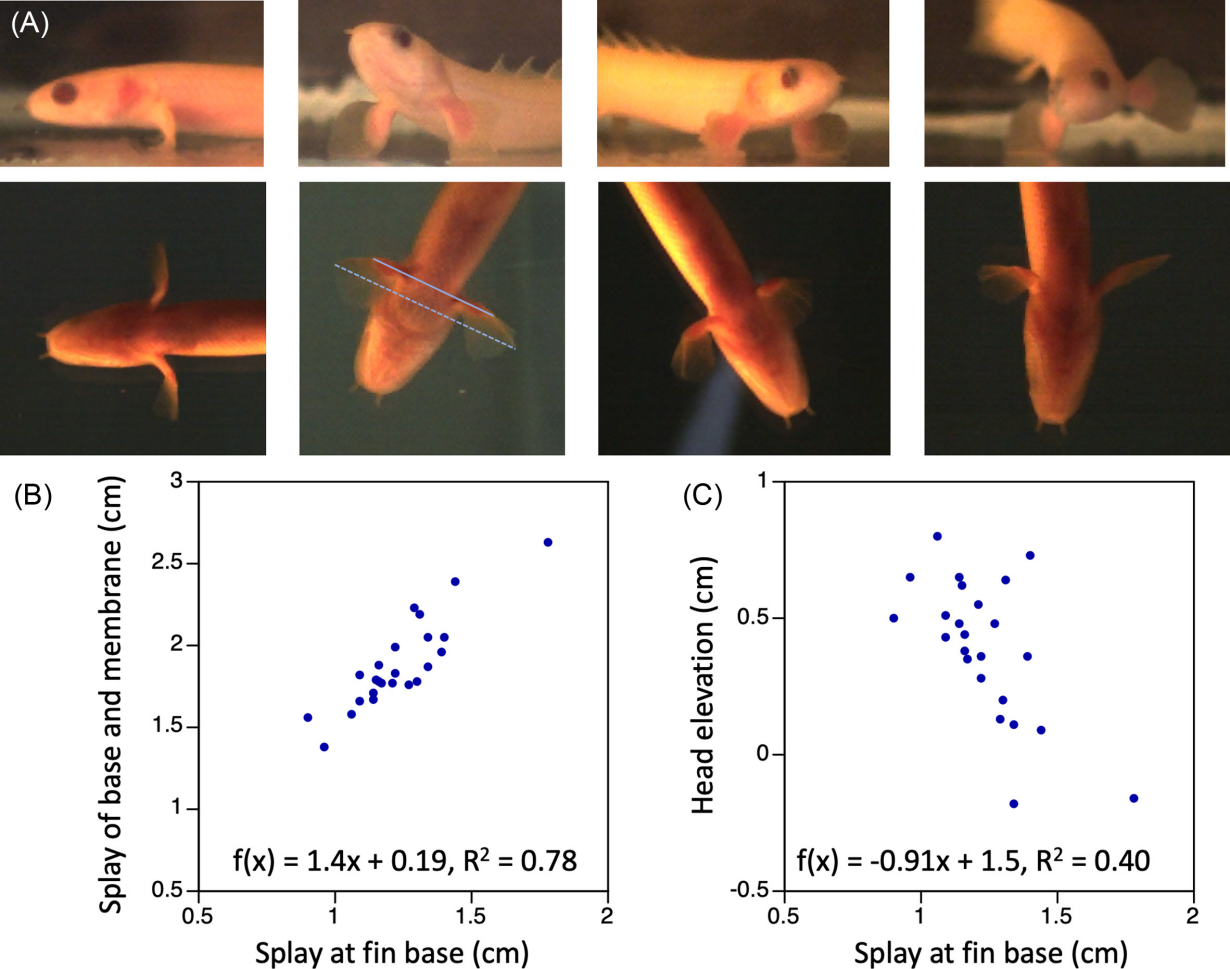
Substrate-based posture of the Senegal bichir, *P. senegalus*. **(A)** Lateral (top row) and ventral (bottom row) views of four postures from one individual bichir, illustrating a range of stable positions that the fish assumed resting on its pectoral fins. For scale, maximum head width = 0.87 cm and head height at the caudal eye margin = 0.58 cm. **(B)** Fin splay increased disproportionately to splay at the fin base. **(C)** Head elevation decreased with increased splay of the fin base. Splay of the fin is the distance between the lateralmost points on the left and right pectoral fins. The span of the fin base is measured from lateralmost points at the proximal end of the fin membrane on the left and right fins. As examples, the dashed line on ventral view in (**A**) indicates the splay of the full fin and the solid line indicates the splay of the fin base

Senegal bichirs regularly perched on the bottom of the filming tank. Pectoral fins extended below the body but at a range of angles (Fig. 5). In nearly all trials (14 of 16), the fins fanned forward toward the lateral aspect of the fin, in contrast to the fin bending observed in the regionalized fins. The fins splayed slightly to the sides below the body with the distance from left to right fin lateralmost tip of the fin calculated at the points most distant from the body averaging 1.72 cm ± 0.34 cm (or 0.26 bl ± 0.05 bl). Regression of splay of the whole fin against splay at the base, f(x) = 1.4x + 0.19, *R*^2^ = 0.78 shows a positive slope that we attribute, at least in part, to the shape of the fin membrane, which is shorter at each end where it meets the fin base and longer in the middle; increasing splay of the base, thus would disproportionately increase splay of the pectoral fins. One question about bichir pectoral fin use during posture was whether, like the hawkfish, they showed variability in splay that would suggest a mechanism for postural modulation. The regression of head elevation to splay of the fin base was f(x) = -0.91x + 1.5, *R*^2^ = 0.40, suggesting that they may vary the height of the head above the ground by changing the angle of the fin to the side of the body. However, there was also considerable variation in the positioning of the membrane and contact with the bottom, including, in some cases, propping on the tips of the fins (Fig. 5). Unlike the other substrate-dependent fish reviewed here, bichirs have a gas bladder, which they fill by gulping air at the surface, providing an alternative approach to lift. It is unclear how buoyancy might impact fin use in substrate-based posture. One example in Fig. 5 is of a bichir that perched on one fin stably: we have seen no indications of hawkfish or round goby with like capabilities.

## Conclusions

The ability of animals to use their limbs for behavior in different physical environments can provide both insight into the evolution of multifunctionality and alternative approaches to design. Here, we focused specifically on pectoral fin adaptations associated with substrate use in posture in relation to more typical function in swimming. We highlight the importance of considering fin sensory function in the examination of multifunctionality and fin regionalization. Adaptation of the sensory system is not as readily examined as system-level features of the skeleton and muscles but is key to function. Comparisons between dorsal and ventral parts of the regionalized fin or between generalized fins that are and are not used in substrate-based posture provide an opportunity to examine the sensory specialization of aquatic vertebrate fins and their similarities to such specialization in tetrapod arms and legs.

With regionalization of the fin and substrate-based posture, the use of the pectoral fin to beat rhythmically in swimming appears to become less common. One hypothesis to explain this is that the adaptations of the pectoral fin for station holding on the bottom decrease its performance in rhythmic, fin-based swimming, and the responsibility for locomotor force generation shifts entirely to the body axis and caudal fins. Alternatively, the negative buoyancy of most substrate associated species may create the need for lift generation and perhaps selective pressure drives the pectoral fin toward this specialized function in swimming. We show that pectoral fin swimming is not necessarily lost in regionalized fins; hawkfish provide an unusual example of a pectoral fin with an extremely specialized ventral fin region used on substrate and dorsal membranous fin region that beats during swimming. Expanded study of the substrate-associated centrarchiforms would be useful in assessing whether this is a unique feature of hawkfishes, or the dwarf hawkfish, or characteristic of pectoral fin regionalization in the centrarchiforms and other orders.

Comparison between the generalized and regionalized fins in the examples discussed here raises two key differences in surface contact mechanisms. First, while free rays of the regionalized fins tended to bend back toward the medial side of the fin, the generalized fins we reviewed typically bent laterally, extending anteriorly. Petersen and Ramsay (2020) describe asymmetry in the structure of the sea robin hemitrichia that relates to its medial bending; it would be interesting to assess these features more broadly across species. Bending the rays under, toward the medial surface of the fin, is conducive to gripping as the fin curves toward the substrate. It may be that soft, generalized fins do not have the ray structure and mechanical properties to allow them to grip and spreading the membrane forward toward the lateral surface results in higher performance station holding when gripping is not possible. Second, the fish with generalized fins appear to use a much broader swath of the fins distal edge to contact the substrate than do fish with regionalized fins. This perhaps augments the performance of the soft fin in gripping. The bichir, with its extended fin base and swim bladder, is unusual among the substrate-dependent fishes. Examining additional teleost groups to build on data in gobies would help to address these issues. We have been assuming that a major function of both types of fins is holding position on a surface and resistance to being dislodged by flow or other means. It is likely that substrate postures have other functional roles that influence the specifics of this surface interaction and related anatomy.

## Methods

### Phylogenetic mapping

To determine whether substrate-associated regionalization was a primitive characteristic or evolved independently in the Centrarchiformes and Perciformes, we reviewed images of all taxa in these two groups that were used in a recent phylogenetic tree (Betancur et al. 2017). Phylogenetic trees were pruned and viewed using FigTree version 1.4.1 (Rambaut 2012), and the packages ape (Paradis et al. 2004), GEIGER (Harmon et al. 2008) and phytools (Revell 2012) in R version 3.3.1 (R Core Team). Using the 'ace' function in the R package ape (Paradis et al. 2004), ancestral character states were estimated across the phylogeny using the equal rates transition rate model to plot character trait maps at the species tips and ancestral reconstructions at the nodes.

We removed species for which clear images of fin anatomy (two views per species from two sources) could not be found from reputable online sources such as FishBase, The Florida Museum of Natural History, and The Fishes of Australia. A single feature 'ventral regionalization' was mapped onto the tree (Fig. 2) with three states: (1) no/minimal ventral regionalization, (2) moderate loss of membrane in association with ventral rays, and (3) extreme/full loss of membrane such that there are ventral free rays. Because inspection of images is nowhere near as precise as visual inspection, we were conservative in mapping and this exploration should be taken as an overview. Subtle features of regionalization could not be assessed nor is part of our conclusions.

*Hawkfish*: all housing and experimental procedures were conducted following University of Chicago Institutional Animal Care and Use Committee guidelines. Hawkfish (*N* = 4; total length range average ± SD: 7.46 ± 0.19 cm, range: 7.2–7.6 cm) were housed individually in 20-gallon tanks maintained at ∼23°C with a natural light/dark cycle. Kinematics were determined two trials per fish and two-three fin beats per trial. One fish was euthanized for anatomical imaging. Filming occurred in the same room as animal housing to limit transport and for similar water temperatures and other conditions. Fish were filmed in a small glass tank (working area 14.5 cm × 20 cm × 15 cm) filled to 10 cm. A total of two fiber optic gooseneck lamps were used to light the working area, augmenting natural light. Fish were left for 5 min to acclimate before trials were conducted. In between trials, lights were removed, and an aerator was placed in the tank. Fish were filmed at 250 fps using three synchronized Photron (Photron, San Diego, CA) high-speed cameras with a 1280 by 1024-pixel spatial resolution (Models: FASTCAM UX-50 160 K-C-8 GB). A total of two of the cameras were positioned at an offset angle to record lateral views, and the third camera was positioned to record a ventral view reflected through a mirror angled at 45° below the tank. The Dwarf Hawkfish are not active swimmers, preferring to perch at the corners of the tank. To prompt swimming and perching away from tank edges, we touched the fish with a plastic pipette. Recordings were carried out and saved using Photron Fastcam Viewer software . Recordings were analyzed every fourth frame (so at 16 ms intervals).

Timing and angular measures were recorded from ventral view video, using lateral views for qualitative confirmation. Fin beat cycles are defined as the duration of peak abduction of the leading edge of one cycle to peak abduction of the leading edge of the next cycle. Peak abduction was a more reliable time marker than adduction as the fin immediately begins adduction once it has reached peak abduction but could remain adducted for much longer. All timing and angle data was taken from ventral view video with lateral views being used to confirm assessment of fin movement in lateral view. The angles of the pectoral fin's leading edge, the ventralmost ray in the dorsal region (ray 8) and the ventralmost ray of the fin (ray 14) were recorded for the left fin using ImageJ (Schneider et al. 2012) (ver: 2.0.0-rc-69/1.52p). As the distal end of some rays are curved for parts of the fin beat, angles were taken using the proximal ray, which remained straight throughout the trial, thus the angle represents the angle of the ray to the body at its base. On the fin, the length of the segment was approximately one half of the length of the ray. Given that the fin is moving in 3D and there are no landmarks on the rays, the end point was imprecise but also not impactful to the angle measured, given that the proximal ray is straight. The other segment used to create the angle, connecting at the base of the fin, was drawn using the margin of the body along the trunk, approximately to the end of the region of the trunk adjacent to the pectoral fin. For the example of axial bending in   Fig. 3, the angle shown represents the angle at the anus, the border of the trunk and tail, of caudal bending, based on segments from the snout tip to the anus and anus to the dorsal posterior tip of the caudal fin. The body is straight at a zero angle and the angle changes with combined tail and caudal fin movement to the right and left sides of the midline.

To assess the anatomy of superficialis muscles and their relation to ventral free rays in the hawkfish, a euthanized experimental animal was dissected fresh and photographed on a Leica (Leica Microsystems (Wetzlar, Germany) MZFL*III* dissecting microscope and with an Olympus (Tokyo, Japan) DP72 camera and cellSens software.

*Bichirs:* to examine underwater posture of the Senegal bichir and compare it to underwater swimming, we recorded and analyzed trials (four fish, four trials per fish, per behavior) of both behaviors using the filming apparatus and methods described for hawkfish. The small bichirs (for total body length was average 66.5 ± 4.7 mm, range: 61.5–72.0 mm) were notably close to the size used by Standen et al. 2016 (64.6 mm average body length). Bichirs were purchased from aquarium suppliers and were captive-bred albino strains.

Analysis of swimming and posture were performed with ImageJ. For swimming, in ventral view peak fin adduction and abduction angles were measured as the angle of the fin region proximal to the fin membrane to the side of the body posterior to the fin. Both the medial edge of the proximal fin region and the side of the body visible in ventral view were straight, simplifying measurement. Cycle duration was measured as the duration between successive peak abductions. Lateral views provided helpful qualitative confirmation, but fin strokes were not digitized in lateral view.

## Supplementary Material

icac061_Supplemental_File

## Notes

From the symposium “Lesser known transitions: organismal form and function across abiotic gradients’’ presented at the annual meeting of the Society for Integrative and Comparative Biology virtual annual meeting, January 3–February 28, 2022.
